# Molecular analysis of *Baylisascaris columnaris* revealed mitochondrial and nuclear polymorphisms

**DOI:** 10.1186/1756-3305-6-124

**Published:** 2013-04-29

**Authors:** Frits Franssen, Kayin Xie, Hein Sprong, Joke van der Giessen

**Affiliations:** 1 National Institute for Public Health and Environment (RIVM), Center for Zoonoses and Environmental Microbiology (cZ&O), Antonie van Leeuwenhoeklaan 9, P.O. Box 1, Bilthoven 3720 BA, The Netherlands

**Keywords:** *Baylisascaris columnaris*, Molecular characterization, Species-specific sites

## Abstract

**Background:**

*Baylisascaris* species are intestinal nematodes of skunks, raccoons, badgers, and bears belonging to the genus Ascarididae. Oral uptake of embryonated *Baylisascaris* sp. eggs by a wide variety of mammals and birds can lead to visceral, ocular and neurological larva migrans. *B. procyonis*, the raccoon roundworm, is known to cause severe illness in intermediate hosts and in humans, whereas the skunk roundworm *B. columnaris* is probably less pathogenic. Skunks and raccoons are kept as pets in Europe, sometimes together with cats and dogs, living in close contact with humans. *B. procyonis* and *B. columnaris* are difficult to differentiate based on morphological criteria and molecular and phylogenetic information concerning *B. columnaris* is missing. This is the first study on the genetic characterisation of *B. columnaris*, based on mitochondrial and nuclear molecular markers.

**Methods:**

*B. columnaris* worms were isolated from pet skunks, and used for molecular analysis. PCR primers targeted at mitochondrial cytochrome c oxidase 1 and 2 (CO1 and CO2), ribosomal ITS1-5.8S-ITS2 and ribosomal 28S genes were used. DNA sequences from *B. columnaris, B. procyonis* and *B. transfuga* from bears were analysed by cluster analysis.

**Results:**

Four different multi-locus genotypes were found in *B. columnaris,* based on 14 single nucleotide polymorphisms (SNPs) and two insertions / deletions in CO1, CO2, ITS1-5.8S-ITS2 and 28S.

**Conclusions:**

The genetic characteristics of *B. columnaris* show close resemblance to those of *B. procyonis*, but in contrast to *B. procyonis,* show several polymorphisms in both mitochondrial and nuclear markers. These polymorphisms could be used as a tool to differentiate *B. columnaris* from *B. procyonis* in molecular diagnostic assays, and to identify *B. columnaris* by PCR, in addition to or replacing morphometric analysis. This might lead to more insight into the zoonotic relevance of *B. columnaris* in humans.

## Background

*Baylisascaris* species in raccoons (*B. procyonis*), skunks (*B. columnaris*) and badgers (*B. melis*) cause visceral, ocular and neural larva migrans (VLM, OLM, NLM) in a range of intermediate hosts, which serve as prey.

In the USA, the raccoon parasite *B. procyonis* is considered the most common cause of larva migrans syndrome in a wide range of intermediate host species and humans [1,2]. In Germany, several cases of clinical baylisascariosis have been described in animal caretakers and in a boy owning a pet raccoon that was kept indoors [3-5]. There are over twenty well-documented cases of OLM and severe or even fatal neurological disorders in humans caused by *B. procyonis*, especially in children and young adults exhibiting pica or geophagy [1,6].

Skunks are a definitive host of *B. columnaris*, which like *B. procyonis*, causes VLM, OLM and NLM in naturally or experimentally infected rodents and rabbits, both prey of skunks [7-9]. Experimental infections showed that *B. procyonis* is more pathogenic to mice than *B. columnaris,* due to faster growth to 1 mm larval size, which correlates with the first observation of nervous symptoms in this host species [10]. Moreover, 2–5 larvae of *B. procyonis* in a single mouse brain lead to death within 25 days post infection (pi), whereas mice with 2–5 *B. columnaris* larvae in their brain died between 20 and 50 days pi or later [10]. However, administration of high numbers of *B. columnaris* eggs evokes the same clinical symptoms as are seen with *B. procyonis* in lower numbers [2]. Skunks and raccoons are kept as pets in Europe, sometimes together with cats and dogs, living in close contact with humans. Dogs can act as both paratenic and definitive host for *B. procyonis*[11], but no experimental or natural infections of cats with *B. procyonis* were documented [2,12].

Diagnosis in the final host is based on morphometric identification of *B. procyonis* and *B. columnaris* individual worms or faecal eggs, although identification to species level is often hampered by diversity in size and developmental stage and the vast majority of worms being female.

Diagnosis of larva migrans caused by ascarid nematodes in humans mainly depends on serological or histochemical assays, which often have a low level of specificity. Additional serological techniques like western blot and recombinant antigen-based BpRAG1 ELISA showed higher ability to discriminate between *B. procyonis* and *Toxocara canis* in patients with larva migrans syndrome [13,14], but tools to discriminate between *Baylisascaris* species are not available, which makes it difficult to assess the potential public health relevance of *B. columnaris*.

Molecular studies have been published concerning mitochondrial and nuclear markers of *B. procyonis*, *B. schroederi* and *B. transfuga*[15-18]. The phylogenetic relationship between ascarid nematodes originating mainly from wildlife host species has been studied, including *B. procyonis*, *B. schroederi*, *B. transfuga* and *B. ailuri*[19]. Analysis of 12 protein encoding genes showed that *B. procyonis* is closely related to, but distinct from other *Baylisascaris* species (*B. ailuri*, *B. transfuga* and *B. schroederi*) [20], but *B. columnaris* was not included in any of these studies.

In this paper, we studied nuclear and mitochondrial markers, based on the mitochondrial genes cytochrome c oxidase 1 and 2 (CO1 and CO2) and the ribosomal ITS1, ITS2, 5.8S and 28S genes of *B. columnaris* and *B. procyonis*. We show the close genetic relationship between these two *Baylisascaris* species and in addition, we identified four different multilocus types of *B. columnaris*, based on 14 single nucleotide polymorphisms (SNPs) in aforementioned genes and differences in G-A tandem repeats in ITS2. Based on these results, we conclude that *B. columnaris* and *B. procyonis* are closely related, but can be discriminated. Therefore, tools can be developed to study the potential zoonotic relevance of *B. columnaris* in humans in more detail.

## Methods

### Animals and parasites

From 2011 to 2012, parasites that were expelled from skunks after pyrantel pamoate treatment were collected at privately owned shelters in The Netherlands. The collected parasites were sent to our institute in 70% ethanol and identified morphologically using taxonomic characteristics as defined previously [21,22]. Subsequently, a small part (2–4 mm) from the mid-section of each parasite was isolated and transferred to 2 ml tubes containing 0.1 mm silica beads (Lysis matrix B, MP Biochemicals, Eindhoven, The Netherlands). These samples were stored at −20°C until further use.

Dr. Kevin Kazacos (Purdue University, West Lafayette, Indiana, USA) kindly provided eight ethanol-preserved *B. procyonis* worms from North American raccoons. Dr. Rebecca Davidson (Norwegian Veterinary Institute, Oslo, Norway) kindly provided twenty-three *Baylisascaris* worms from raccoons kept in Norway*.* Dr. Herman Cremers (Utrecht University, The Netherlands) provided *B. procyonis, B. columnaris* and *B. transfuga* adult worms. Additionally, *B. transfuga* parasites were isolated from a brown bear and a sloth bear, both zoo animals. Details on the parasites used in this study are shown in Table 1.

**Table 1:** ***Baylisascaris *worm origin and gender**

**Species**	**#Worms**	**Male**	**Female**	**Juvenile**	**Host name**	**Host gender**	**Source**
*B. columnaris*	19	5	10	4	S and/or I^1^	M/F^2^	Shelter H (NL)
*B. columnaris*	1	-	-	-	J	M	Shelter K (NL)
*B. columnaris*	5	3	2	0	DD	M	Shelter K (NL)
*B. columnaris*	10	2	5	3	N	F	Shelter K (NL)
*B. columnaris*	31	11	20	0	V	M	Shelter K (NL)
*B. columnaris*	4	0	4	0	P	F	Shelter P (NL)
*B. columnaris*	19	1	18	0	C	F	Shelter P (NL)
*B. columnaris*	30	11	19	0	A	F	Shelter P (NL)
**Totals:**	**119**	**33**	**78**	**7**	**-**	**-**	**-**
*B. procyonis*	8	2	6	0	ns	ns	(USA)
*B. procyonis*	4	1	3	0	652	ns	(Norway)
*B. procyonis*	7	2	5	0	653	ns	(Norway)
*B. procyonis*	10	3	7	0	654	ns	(Norway)
*B. procyonis*	2	0	1	1	655	ns	(Norway)
**Totals:**	**31**	**8**	**22**	**1**	**-**	**-**	**2**

^1^ Origin of worm uncertain due to lack of animal isolation possibilities; these worms were excluded from further molecular analyses. ^2^ Gender of S: male, I: female. ns: not stated.

### DNA isolation

DNA was extracted from worm tissue using the Qiagen DNeAsy Blood & Tissue Kit (Qiagen Benelux BV, Venlo, The Netherlands) according to the manufacturer’s instructions. The protocol was slightly modified by addition of a tissue disruption step before and after proteinase K incubation, using a bead-beater for 45 s at 6.5m/s^2^ (Fastprep™ FP 120, Thermo Electron Corporation, Milford, MA, USA). The isolated DNA was eluted in 10 mM Tris–HCl and stored at −20°C until further use.

### PCR primers

A primer pair targeted at the mitochondrial CO1 was used according to Bowles et al. (1992) [23]. Furthermore, primers targeting the CO2 gene were designed based on the consensus sequence of *B. columnaris* [Genbank accession number FJ357429], *B. procyonis* [AF179908] and *B. transfuga* [AF 179909, HM594949 and FJ890507].

Primer pairs targeted at ITS1-5.8S-ITS2 ribosomal DNA were designed based on the ribosomal consensus sequences of *B. transfuga* [JN617990, AB571304], *B. schroederi* [JN210911, JN210912] and *B. procyonis* [AB053230, AJ007458]. PCR primers for 28S rDNA were designed based on the consensus sequence of *B. transfuga* [HM594950, HM594951 and U94754] and *B. procyonis* [U94753]. Primer sequences are shown in Table 2.

**Table 2:** Overview of the PCR primers used in this study

**Primer name**	**Primer sequence**
CO1 F	5′-ttttttgggcatcctgaggtttat-3′
CO1 R	5′-taacgacataacataatgaaaatg-3′
CO2 F	5′-aattttaattgtagtcttttgtttgg-3′
CO2 R	5′-ctatgattagcaccacaaatc-3′
ITS1-5.8S-ITS2 F	5′-atagtgagttgcacactaatgt-3′
ITS1-5.8S-ITS2 R	5′-ttatatgcttaaattcagcggg-3′
ITS2 F	5′-gccatttatgaattttcaacatgg-3′
ITS2 R	5′-agttatatgcttaaattcagcgg-3′
28S rDNA F	5′-cgaggattcccttagtaact-3′
28S rDNA R	5′-tcggataggtggtcaacg-3′

Primer sequences for PCR amplification of mitochondrial and nuclear markers of *B. columnaris*, *B. procyonis* and *B. transfuga*.

### PCR conditions

All PCR reactions were carried out in 50 μl final volume containing 3 μl genomic DNA, 0.5 μl of each forward and reverse primer (50 μM stock) and 25 μl of Qiagen HotstarTaq Plus polymerase master mix (Qiagen NV, Venlo, The Netherlands). The final reaction volume was adjusted to 50 μl with sterile demineralised water. The PCR amplification of CO1 and CO2 was performed using the following conditions: denaturation at 95°C for 15 min, followed by 35 cycles of 1 min denaturation at 95°C, 1 min annealing at 45°C, 1:15 min elongation at 72°C, followed by a final extension step of 7 min at 72°C.

The PCR amplification of ITS1-5.8S-ITS2 and 28S rDNA was performed under the following conditions: 95°C for 15 min, followed by 40 cycles of 95°C for 30 s, 50°C for 30 s, 72°C for 1:15 min and 72°C for 7 min. PCR products were analysed on 1.5% agarose gel.

### DNA sequencing of amplified PCR products

PCR amplicons were purified using standard procedures (ExoSAP-IT®, Affymetrix, Cleveland, Ohio, USA). Sequence PCR reactions on both strands were carried out in 20 μl final volume containing 3 μl of amplicate, 7 μl sequence buffer, 1 μl of Big Dye Terminator and 1 μl of each PCR primer. The sequence PCR was performed under the following conditions: 95°C for 1 min, followed by 25 cycles of 96°C for 10 min, 50°C for 5 min and finally 60°C for 4 min. Trace files of sequences were generated on an automated ABI sequencer at the Institute’s sequence facility.

### Sequence analysis and phylogeny

Obtained DNA sequences were aligned, analysed with BioNumerics version 6.6 (Applied Maths NV, Sint-Martens-Latem, Belgium) and compared with DNA sequences present in Genbank, after subtraction of the primer sequences. Phylogenetic analysis was performed by Maximum Likelihood inference using 500 bootstraps [24,25].

## Results

### Animals and parasites

**Figure 1:**
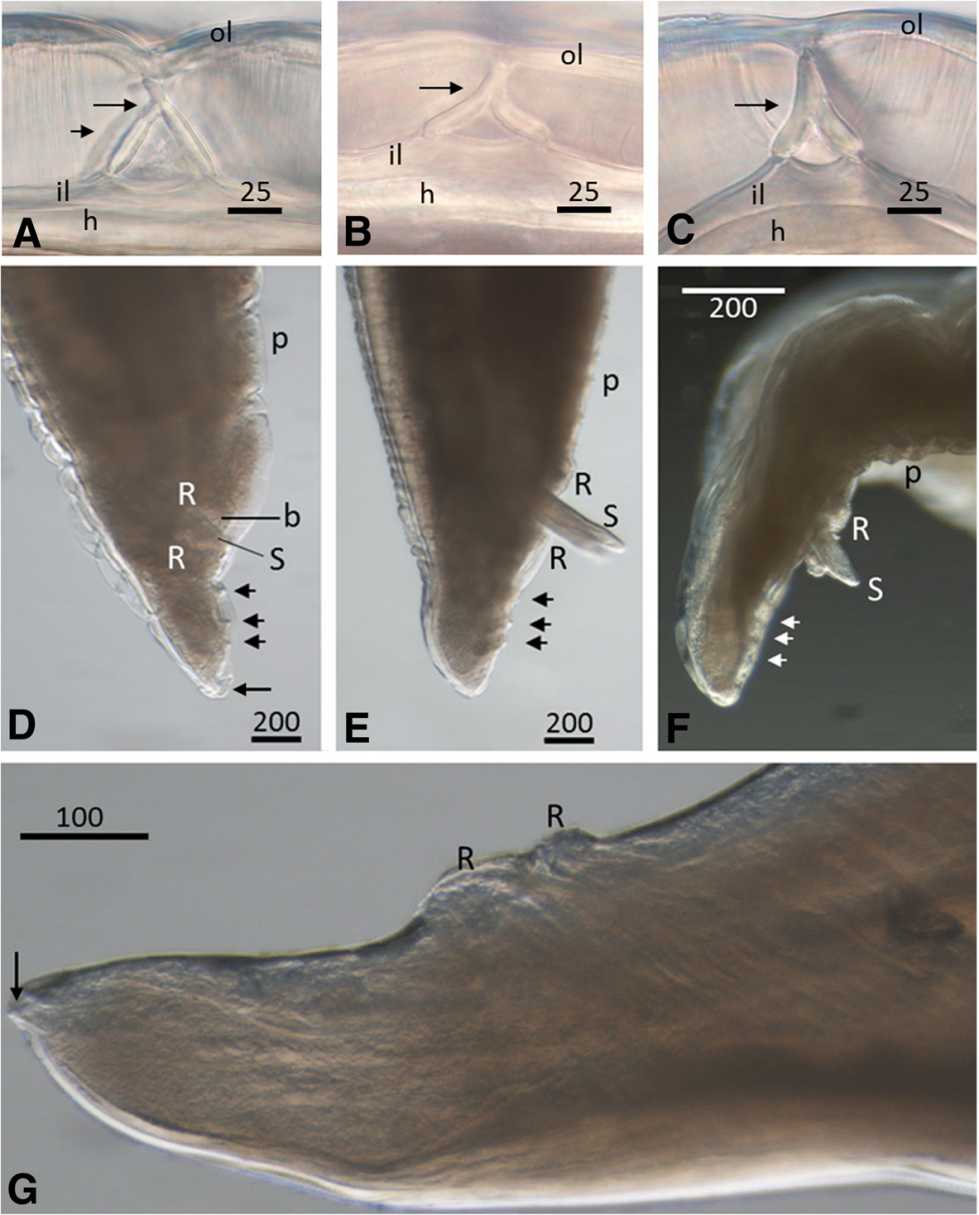
**Morphological differentiation of *Baylisascaris procyonis *(A, D) from *B. columnaris *(B, E and C, F). A** - **C**: Unstained cross section of lateral cuticle through anterior end of adult female worms (about 10 centimetres in length), 6 millimetres behind mouthparts and rotated 90° in plane. Cross section in lateral view showing outer cuticle lining (ol), inner cuticle lining (il), hypodermis (h) and cervical support in hyaline layer (arrow). Sometimes, cervical supports of the deeper layer are visible, though not in focus (arrowhead). **A**: Female *B. procyonis* (isolate Bp9), wide arch-like cervical support **B**: Female *B. columnaris* (K19), narrow A-like support **C**: Female *B. columnaris* (K10), narrow A-like support. **D** - **F**: Posterior end of males showing pre-cloacal papillae (p), pericloacal roughened areas (R) with rounded posterior margin in figure **E**, bare pre-cloacal rim (b), extruded spicules (S, not in focus in **D**), post- cloacal papillae (short arrow), terminal part of tail knob (**D**) or spike (**G**; **E** and **F** out of focus) shaped (long arrow). **D**: Ventrolateral view of *B. procyonis* (Bp19), precloacal roughened patch 42 μm and postcloacal patch 72 μm in width. **E**: Lateral view of *B. columnaris* (K23), **F**: Lateral view of *B. columnaris* (K22). **G**: Lateral view of posterior end of male *B. columnaris* (P27), showing spike shaped posterior end of the tail (arrow) and pericloacal roughened patches in close up; precloacal patch (right) 63 μm and postcloacal patch (left) 79 μm in width. Scale bars in μm. See Table 3 for molecular classification of isolate numbers.

In total, 119 *B. columnaris* worms were isolated from seven skunks (Table 1). Among these are five stray animals (D, N, V, C and A). Worm body size varied, ranging from 3–4 centimetres for adult males and young females, to a maximum of 14 centimetres for adult females. All worms were morphologically identified as *Baylisascaris* sp*.,* based on the absence of cervical alae in adult worms, the morphology and size of intra-uterine eggs (76.7 ± 5.1 by 64.6 ± 5.8 μm, n=10) that were isolated from gravid females, the morphology and size of faecal eggs (72.5 ± 4.1 by 63.2 ± 7.5 μm, n=10) in coprological examination, the typical ascarid mouthparts with three developed lips (one dorsal and two subventral), the presence of precloacal genital papillae, two spicules and perianal roughened patches in adult males. Species identification was carried out using differentiating features as defined by Sprent (1968) [22], which can discriminate between different *Baylisascaris* species. Figure 1 shows characteristic morphological features of *B. procyonis* (A and D), and *B. columnaris* (B, E and C, F) isolates of this present study. Figure 1G zooms in on typical male *B. columnaris* features, being perianal roughened patches of more or less equal width and the spike-shaped terminal end of the tail.

### PCR and sequence analysis

Mitochondrial CO1 amplicons (413 bp) were obtained from forty-three individual *B. columnaris* isolates, nineteen *B. procyonis* and two *B. transfuga* isolates. CO2 amplicons measured 483 bp and were obtained from thirty-eight individual *B. columnaris* isolates, ten *B. procyonis* and one *B. transfuga* isolate. CO2 sequence alignment of fifteen *B. columnaris* isolates (represented by Figure 1C and 1F) revealed 100% CO2 sequence homology with a partial *B. columnaris* CO2 sequence present in Genbank. This sequence was derived of a parasite that was isolated from a road-killed skunk from Indiana, USA [FJ357429]. The other twenty-three *B. columnaris* isolates (represented by Figure 1B, 1E and 1G) showed 99.4% similarity with both *B. columnaris* [FJ357429] and *B. procyonis* [JF951366].

Alignment of the 413 bp CO1 sequences revealed six single nucleotide polymorphisms (SNPs), three of which were homologous between *B. columnaris* isolates, but differed from *B. procyonis* (nt positions 61, 91 and 259) (Table 3). Alignment of the 483 bp CO2 sequences showed four additional SNPs, one of which was homologous between *B. columnaris* isolates but different from *B. procyonis* (nt position 66) (Table 3). *In silico* translation of the open reading frames of CO1 and CO2 resulted in 137 and 161 amino acids without stop codons respectively.

**Table 3:** **Multi-locus types of *Baylisascaris columnaris *mitochondrial genes**

**Worm**	**Worm**	**Skunk**	**CO1 fragment**						**CO2 fragment**				**ML**
**isolates**	**gender**	**ID**	**59**	**61**	**79**	**91**	**229**	**259**	**66**	**117**	**280**	**480**	**type**
K14, K16	F	N	T	G	C	A	G	G	G	A	C	G	I
K22, K24	M	V	T	G	C	A	G	G	G	A	C	G	I
K15, K10	F	N	T	G	C	A	G	G	G	A	C	G	II
K21	M	V	T	G	C	A	G	G	G	A	C	G	II
K17	F	V	C	G	T	A	A	G	G	G	T	T	III
K23	M	V	C	G	T	A	A	G	G	G	T	T	III
P24	F	P	C	G	T	A	A	G	G	G	T	T	III
P27	M	A	C	G	T	A	A	G	G	G	T	T	III
K19, 20	F	V	C	G	T	A	A	G	G	G	T	T	IV
*B. procyonis* isolates			T	A	T	G	A	A	A	A	T	T	-
*B. transfuga* isolates			T	A	T	G	T	G	G	A	T	G	-

Four different genotypes of *Baylisascaris columnaris* were found: two variants of mitochondrial genes CO1 and CO2, and two variants of nuclear genes ITS1-5.8S-ITS2 and 28S rDNA. These variants were linked two by two. Neither recombinations of mitochondrial type 1 and 2, nor recombinations of nuclear type 1 and 2 occurred in individual worms. No preference of any combination for worm gender was observed.

**Figure 2:**
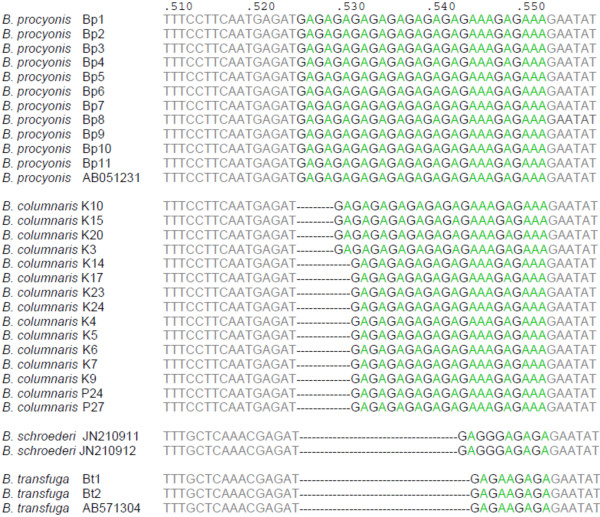
**G-A tandem repeats with different length on the ITS2 gene.***B. procyonis* has 9 G-A tandem repeats on the ITS2 gene, where *B. columnaris* has two tandem repeats of different length, six or seven G-A repeats. *B. transfuga* has a much shorter G-A tandem repeat sequence, which is one nucleotide shorter and different from the tandem repeat in *B. schroederi*.

Ribosomal ITS1-5.8S-ITS2 amplicons (644 or 647 bp) were obtained from fifteen *B. columnaris* isolates, twelve *B. procyonis* isolates (650 bp) and two *B. transfuga* isolates (630 bp). Sequence alignment revealed an insertion as compared to *B. procyonis* in the ITS1 gene (nt 1–228) at nucleotide position 152 and a SNP at nt 201. The sequence of 5.8S rDNA (nt 229–385) was homologous between *B. columnaris* and *B. procyonis* isolates, but differed from *B. transfuga* (nt 230, 337 and 346) (Table 4). In the ITS2 gene region (nt 386 and further), a tandem repeat was found commencing at nucleotide position 526 and containing a number of G-A inserts varying with *Baylisascaris* species. Nine G-A tandem repeats were identified for *B. procyonis*, which was corroborated by comparison with Genbank accession number AB051231. Two genotypes were found in *B. columnaris*: one with six G-A tandem repeats and another with seven. *B. transfuga* had only two G-A tandem repeats, which was corroborated by Genbank accession number EU642819, and was different from the tandem repeat in *B. schroederi* [JN210911 and JN210912] (Figure 2).

**Table 4:** **Multi-locus types of *Baylisascaris columnaris *ribosomal genes**

**Worm**	**Worm**	**Skunk**	**ITS1**		**5.8S**			**ITS2**	**28S**		**ML**
**isolates**	**gender**	**ID**	**152**	**201**	**230**	**337**	**346**	**527**	**49**	**487**	**type**
K14, K16	F	N	-	T	G	T	C	6	C	G	I
K22, K24	M	V	-	T	G	T	C	6	C	G	I
K15, K10	F	N	A	T	G	T	C	7	C	C	II
K21	M	V	A	T	G	T	C	7	C	C	II
K17	F	V	-	T	G	T	C	6	C	G	III
K23	M	V	-	T	G	T	C	6	C	G	III
P24	F	P	-	T	G	T	C	6	C	G	III
P27	M	A	-	T	G	T	C	6	C	G	III
K19, 20	F	V	A	T	G	T	C	7	C	C	IV
*B. procyonis* isolates			-	C	G	T	C	9	T / C	C	-
*B. transfuga* isolates			A	T	T	C	T	-	C	T	-

The numbers refer to nucleotide positions of the SNP in the gene sequence alignments. For ITS2, the number of G-A tandem repeats from nucleotide position 526 onward is shown. The characteristics of *B. procyonis* and *B. transfuga* are shown for reference. (F: female, M: male, ML: multi-locus).

Nuclear 28S rDNA amplicons (718 bp) were obtained from fifteen *B. columnaris*, eight *B. procyonis* and two *B. transfuga* isolates. Sequence alignment revealed an SNP for *B. procyonis* on nucleotide position 61, (either C or T). A polymorphism at nucleotide position 499 for *B. columnaris* (G or C) was found, which coincided with six or seven G-A tandem repeats on the ITS2 gene sequence (Table 4). Combination of mitochondrial and nuclear sequence data resulted in four multi-locus genotypes (I to IV, Tables 3 and 4).

Sequence data for *B. columnaris*, *B. procyonis* and *B. transfuga* obtained in this study were deposited in Genbank [accession numbers KC543466-KC543471 (28S), KC543472-KC543477 (CO1), KC543478-KC543483 (CO2) and KC543484-KC543489 (ITS1-5.8S-ITS2)].

### Phylogenetic analysis

**Figure 3:**
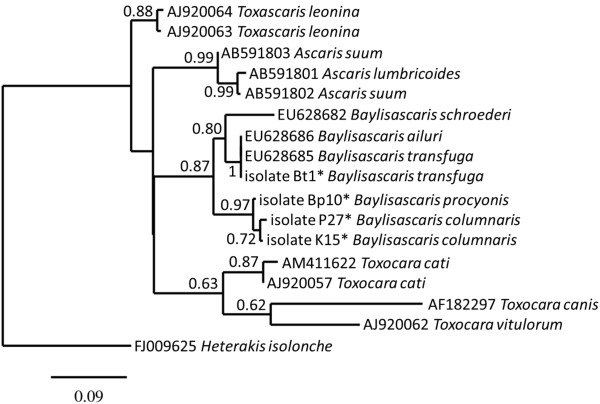
**Relative position of *Baylisascaris *in the genus Ascarididae.** ML of CO1 gene sequences of ascarid nematode species available in Genbank and new isolates (this study). The consensus CO1 gene sequence of the *B. transfuga* isolates shows 100% identity with sequences from Genbank. CO1 gene sequences of *B. procyonis* and *B. columnaris* isolates separate from other ascarid species and from each other with high bootstrap support (500 bootstraps). * This study, see also Figure 2 and Table 3.

To confirm the relative position of *B. columnaris* within the group of ascarid nematodes, a maximum likelihood (ML) tree was inferred from CO1 sequences of *B. procyonis*, *B. columnaris* and *B. transfuga* (this study) and of other ascarid nematodes present in Genbank [AB591801, AB591802, AB591803, AF182297, AJ920057, AJ920062, AJ920064, AJ920063, EU628682, EU628685, EU628686 and HM594948]. A CO1 sequence of *Heterakis isolonche* [FJ009625] was used as out-group (Figure 3).

**Figure 4:**
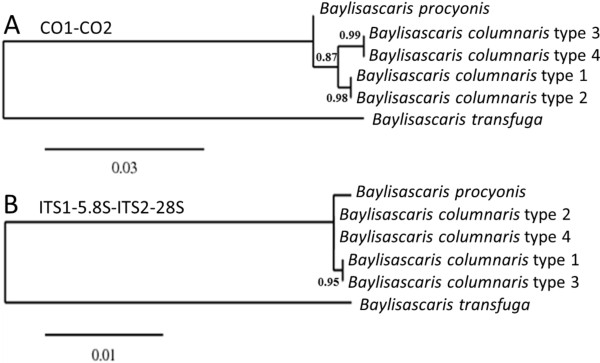
**Maximum likelihood trees inferred from *Baylisascaris *sp. mitochondrial and ribosomal gene sequences. A**: ML of concatenated mitochondrial markers CO1 and CO2 shows a clear separation between *B. procyonis* and *B. columnaris*, which in turn is divided into two different groups. **B**: ML of concatenated ribosomal markers ITS1-5.8S-ITS2 and 28S again shows two *B. columnaris* groups, which separate from *B. procyonis*, although to a lesser extent, due to the number of G-A repeats (9, 7 and 6) as most important difference (see also Table 4).

The inferred ML tree of concatenated CO1 and CO2 sequences shows two *B. columnaris* clusters (type 1 and 2, 15 isolates, and type 3 and 4, 23 isolates), which are separated from *B. procyonis*, supported by high bootstrap values (Figure 4A). The *B. columnaris* intra-species difference based on concatenated CO1 and CO2 sequences was 0.68%, whereas the inter-species differences with *B. procyonis* were 0.71% and 0.91% respectively. An inferred ML tree from concatenated ribosomal markers (ITS1-5.8S-ITS2 and 28S) again revealed two *B. columnaris* groups which diverge from *B. procyonis* (Figure 4B), but are not identical to the ML shown in Figure 4A. However, this is based on only three SNPs and one nucleotide insert, which is too few to obtain a reliable differentiation.

## Discussion

This is the first study showing the close relatedness based on molecular data and phylogenetic analysis of *B. procyonis* and *B. columnaris*, which was isolated from pet skunks. *B. columnaris* is an exotic species in Europe, which is imported together with its final host *M. mephitis*. Evaluation of the public health relevance of *B. columnaris* is hampered by the fact that molecular data and description of clinical manifestations in humans are absent in the literature, despite recent publications concerning molecular characterization of various *Baylisascaris* species [17-20]. So far, only a single (partial) sequence of the mitochondrial CO2 gene of *B. columnaris* was available in Genbank at the time [accession number FJ357429] [26].

The present study corroborates the very close relationship between *B. procyonis* from raccoons and *B. columnaris* from skunks, based on morphological, molecular and phylogenetic analyses.

The combined mitochondrial and ribosomal DNA sequences of *B. columnaris* yielded four multi-locus genotypes, which could be retrieved from one single host animal. Other individual skunks carried either one or two *B. columnaris* multi-locus genotypes. Since we analysed single worms from individual animals, we were able to show the presence of two genotypes of mitochondrial CO1 and CO2, indicating separate maternal lineages and possibly different geographical origins of the *B. columnaris* parasites.

The close relationship with *B. procyonis* both on mitochondrial and nuclear markers, and the relative distance between the two *B. columnaris* mitochondrial variants, initially raised the question whether these genuinely were two genotypes of one species, taking into account that their skunk hosts had artificially been introduced into a new environment and the history of most animals was not known. However, all four multi-locus genotypes could be isolated simultaneously from one individual skunk and the recombination of mitochondrial with nuclear subtypes showed that there is interbreeding between the two phylogroups of *B. columnaris*, supporting that these are indeed one species.

The origin of the distinct genotypes identified in *B. columnaris* are unknown, but may also reflect two (ancient) subpopulations in the skunk host of *B. columnaris* in North America [27]. *B. columnaris* CO2 sequences of both multi-locus sequence type (MLST) I and MLST II are 100% homologous to that of *B. columnaris* isolated from a skunk in Indiana, where 75% of skunks is of the East phylogroup [27].

*B. procyonis* is considered the most common cause of larva migrans syndrome in a wide range of intermediate host species and humans [1,2], and several cases of clinical baylisascariosis have also been described in humans in Europe [3-5]. However, since diagnostic tools are lacking, it is not known whether *B. columnaris* could cause larva migrans in humans. As serological assays do not discriminate between *Baylisascaris* species as mentioned before, the molecular data presented in this study might be used to design molecular diagnostic assays, to evaluate the public health relevance of *B. columnaris*. Moreover, these data might provide tools to monitor introduction and prevalence of these exotic species in wildlife.

## Conclusions

This is the first study presenting molecular data and phylogenetic analysis of *B. columnaris*. The genetic characteristics presented in this study showed a close genetic resemblance of *B. procyonis* and *B. columnaris*. Moreover, the two *Baylisascaris* species can be distinguished using species-specific sites as described in this study, which could be used in PCR-based assays to study the public health relevance of this parasite.

## Competing interests

The authors declare that they do not have competing interests.

## Authors’ contributions

FF was involved in collecting and identifying the nematodes, primer design, molecular lab work, phylogenetic analysis and writing the manuscript. KX was involved in primer design, molecular lab work, analyses of the molecular data and writing the manuscript. HS had an advising role in molecular and phylogenetic analysis and contributed to writing of the manuscript. JvdG arranged funding and initiated the study design, supervised the practical work and contributed to writing of the manuscript. All authors read and approved the manuscript.
